# Deficiency of MTAP Is Frequent and Mostly Homogeneous in Pancreatic Ductal Adenocarcinomas

**DOI:** 10.3390/cancers17071205

**Published:** 2025-04-01

**Authors:** Natalia Gorbokon, Katharina Teljuk, Viktor Reiswich, Maximilian Lennartz, Sarah Minner, Ronald Simon, Guido Sauter, Waldemar Wilczak, Till Sebastian Clauditz, Nina Schraps, Thilo Hackert, Faik G. Uzunoglu, Martina Kluth, Lukas Bubendorf, Matthias Matter, Florian Viehweger, Morton Freytag, Frank Jacobsen, Katharina Möller, Stefan Steurer

**Affiliations:** 1 Institute of Pathology, University Medical Center Hamburg-Eppendorf, 20246 Hamburg, Germany; n.gorbokon@uke.de (N.G.); k.teljuk@freenet.de (K.T.); v.reiswich@uke.de (V.R.); m.lennartz@uke.de (M.L.); s.minner@uke.de (S.M.); g.sauter@uke.de (G.S.); w.wilczak@uke.de (W.W.); t.clauditz@uke.de (T.S.C.); m.kluth@uke.de (M.K.); f.viehweger@uke.de (F.V.); ka.moeller@uke.de (K.M.); s.steurer@uke.de (S.S.); 2 General, Visceral and Thoracic Surgery Department and Clinic, University Medical Center Hamburg-Eppendorf, 20246 Hamburg, Germany; n.schraps@uke.de (N.S.); t.hackert@uke.de (T.H.); f.uzunoglu@uke.de (F.G.U.); 3 Institute of Pathology, University Hospital Basel, 4031 Basel, Switzerland; lukas.bubendorf@usb.ch (L.B.); matthias.matter@usb.ch (M.M.); 4 Pathologie-Hamburg, Labor Lademannbogen MVZ GmbH, 22419 Hamburg, Germany; f.jacobsen@uke.de

**Keywords:** MTAP deficiency, FISH, IHC, tissue microarray, adenocarcinoma of the pancreas

## Abstract

Background: The complete loss of S-methyl-5′-thioadenosine phosphorylase (MTAP) expression, often due to homozygous 9p21 deletion, creates a druggable vulnerability in cancer cells. Methods: A total of 769 primary pancreatic ductal adenocarcinomas were analyzed on tissue microarrays with MTAP immunohistochemistry (IHC) and 9p21 fluorescence in situ hybridization (FISH). Intratumoral heterogeneity was assessed on a “heterogeneity” TMA containing up to nine samples from different areas of 236 primary tumor and nodal metastases, and whole sections of all tumor blocks from 19 cancers. Results: MTAP expression loss was found in 181 (37.9%) of 478 interpretable primary tumors and was unrelated to pT, pN, grade, and tumor size. MTAP expression loss was homogenous in 37.6% and heterogeneous in 1.1% of the 181 tumors, with at least three evaluable samples on the heterogeneity TMA. On whole sections, 1 of 19 tumors showed heterogeneous MTAP loss. The correlation between IHC and FISH was nearly perfect, with 98.8% of MTAP-deficient samples showing a 9p21 deletion. Conclusions: MTAP expression loss is frequent, caused by homozygous deletion, and mostly homogeneous in pancreatic ductal adenocarcinomas. Considering also their aggressive clinical behavior, pancreatic adenocarcinomas may represent an ideal cancer type for studying new drugs targeting MTAP-deficient cancer cells in clinical trials.

## Simple Summary

Ductal adenocarcinoma of the pancreas is a cancer with exceedingly dismal prognosis as less than 5% of all patients survive more than 5 years. Therefore, targeted molecular therapies are urgently needed. MTAP deficiency is currently one of the most promising molecular targets for tumor therapies. MTAP deficiency—often caused by homozygous 9p21 deletion—is easily detectable by immunohistochemistry because it is characterized by a complete MTAP expression loss. It makes tumor cells more susceptible to PRMT5- and MAT2A-inhibiting drugs as well as to pemetrexed (antifolate therapy). However, the success of targeted therapies critically depends on the extent of heterogeneity of the targeted tumor cell trait in clinical tumors. In this study, intratumoral heterogeneity of MTAP deficiency was thus assessed in more than 200 ductal adenocarcinomas of the pancreas in a tissue microarray format and whole-section analysis was carried out by immunohistochemistry and fluorescence in situ hybridization. MTAP deficiency was found in almost 40% of all cancers, was always caused by homozygous deletion, and was rarely heterogenous in TMA and large-section analyses (less than 10%). Pancreatic adenocarcinomas may thus represent an ideal cancer type for studying new drugs targeting MTAP-deficient cancer cells in clinical trials.

## 1. Introduction

S-methyl-5′-thioadenosine phosphorylase (MTAP) is an essential enzyme within the salvage adenine synthesis pathway [1]. The *MTAP* gene is located at 9p21.3, 30 kb apart from the *CDKN2A* gene, which is deleted in ≤ 15% of all human cancers [2,3,4]. *MTAP* deletions occur in 80–90% of homozygously *CDKN2A*-deleted tumors (co-deletion of both genes) [5]. Deficiency of MTAP leads to critical cancer cell vulnerability towards drugs targeting several different pathways [6]. In MTAP-deficient cancer cells, adenine synthesis can only be maintained by de novo biosynthesis. Therefore, inhibition of enzymes critical for folate synthesis leads to increased apoptosis of MTAP-deficient tumor cells in experimental models and shows anti-cancer efficiency in patients with urothelial bladder carcinomas [6]. Tumor cells with MTAP deficiency can also be successfully targeted by inhibitors of methionine adenosyltransferase II, alpha (MAT2A), and protein arginine N-methyltransferase 5 (PRMT5) (summarized in [7]). PRMT5 regulates the activity of numerous proteins by essential methylation of the target proteins [8]. MAT2A is critically needed for the synthesis of S-adenosylmethionine (SAM), the methyl donor and substrate of PRMT5 [9,10]. As PRMT5 is inhibited by the accumulation of the unprocessed MTAP metabolite MTA in tumor cells [11], MTAP deficiency makes these cells more susceptible to PRMT5- or MAT2A-inhibiting drugs (summarized in [7]). A clinical phase 1 trial published in November 2023 demonstrated a significant reduction in tumor size in patients with MTAP-deficient epithelioid malignant mesotheliomas, non-small-cell lung carcinomas (NSCLC), malignant melanomas, and other adenocarcinomas after treatment with the PRMT5 inhibitor MRTX1719 [12]. Multiple further clinical trials targeting PRMT5 are ongoing [13,14,15].

Tumors that can benefit from targeting MTAP deficiency must be identified by molecular tumor analyses. Immunohistochemistry (IHC) is a suitable method for detecting MTAP deficiency, because MTAP is ubiquitously expressed in normal cells [16,17]. In a previous study evaluating more than 13,000 cancers from 149 different tumor types by MTAP IHC, we identified several MTAP-deficient tumor types including neuroendocrine neoplasms (up to 80% MTAP-deficient), Hodgkin lymphoma (50.0% MTAP-deficient), mesothelioma (up to 37% MTAP-deficient), gastro-intestinal adenocarcinoma (up to 41% MTAP-deficient), urothelial neoplasms (up to 37% MTAP-deficient), and squamous cell carcinomas (up to 38% MTAP-deficient). Furthermore, we identified ductal adenocarcinoma of the pancreas as one of the most common MTAP-deficient cancers, with an MTAP loss rate of approximately 30% [18]. Pancreatic cancer is usually detected at the advanced tumor stage where most therapies are no longer effective. Therefore, the survival rate has hardly changed in the last 60 years. Considering the dismal prognosis of these tumors—less than 5% of patients survive more than 5 years—additional treatments are urgently needed [19]. However, the clinical efficiency of molecular markers critically depends on the extent of heterogeneity of the targeted tumor cell trait in clinical tumors. It must be expected that tumors with heterogeneous MTAP deficiency will be much less responsive to respective therapies than cancers with MTAP deficiency in all cancer cells.

To further evaluate the potential of exploiting the synthetic lethality introduced by MTAP deficiency in ductal adenocarcinoma of the pancreas we expanded our initial cohort for a better assessment of clinicopathological relationships and evaluated the intratumoral heterogeneity of MTAP deficiency in cancers from more than 200 patients.

## 2. Materials and Methods

### 2.1. Tissue Microarray

Our set of primary tumor TMAs contained a series of 769 ductal adenocarcinomas of the pancreas treated by pancreatectomy, of which the specimens were analyzed at the Institute of Pathology of the University Medical Center Hamburg-Eppendorf (Hamburg, Germany) or at the Institute of Pathology at the University Hospital in Basel (Basel, Switzerland). Data on pT and pN category as well as histological grade were taken from the pathology reports (Supplementary Table S1). Follow-up data (overall survival) were available for 123 patients (median: 8 months; range: 1–148 months). From all patients, one tissue cylinder with a diameter of 0.6 mm was taken from one tumor-containing tissue block. An additional pancreatic cancer heterogeneity TMA was constructed from a subset of 236 of our cancer patients (3 pT1, 29 pT2, 150 pT3, and 35 pT4 carcinomas) for which at least 3 different cancer-containing tissue blocks were available. From these patients, 6 spots per primary tumor and 1 spot each per nodal metastasis (maximum: 3 spots from 3 metastases) in 112 nodal-positive patients were arrayed. The 6 samples per primary tumor were taken from as many as possible different blocks and as far away from each other as possible. For each TMA construction, a 0.6 mm-diameter tissue core was taken. In total, the heterogeneity TMA included 1578 tissue cores from 236 patients. All tissues were fixed in 4% buffered formalin and then embedded in paraffin. A homemade semiautomated precision instrument was used for tissue arraying [20]. Utilization of archived diagnostic leftover tissues for the manufacturing of tissue microarrays and their analysis for research purposes as well as for patient data analysis has been approved by local laws (HmbKHG, §12.1) and by the local ethics committee (Ethics Commission Hamburg, WF-049/09). All work has been carried out in compliance with the Helsinki Declaration.

### 2.2. Fluorescence In Situ Hybridization (FISH)

FISH was executed as previously described [21]. In brief, five-micrometer TMA sections were deparaffinized, rehydrated, and exposed to heat-induced denaturation for 10 min in a water bath at 99 °C in P1 pretreatment solution (Agilent Technologies, Santa Clara, CA, USA; #K5799). For proteolytic treatment, slides were added to VP2000 protease buffer (Abbott, Chicago, IL, USA; #2J.0730) for 200 min at 37 °C in a water bath. A commercial FISH probe kit containing both a 9p21 probe, including the *CDKN2A* and *MTAP* genes, and a centromere 9 probe were utilized for 9p21 copy number detection (ZytoLight^®^ SPEC *CDKN2A*/CEN 9 Dual Color Probe, Zytovision, Bremerhaven, Germany; #Z-2063). Hybridization was carried out at 37 °C overnight. Post-hybridization washes were performed at 37 °C according to the manufacturer’s instructions. Stained TMA spots were manually interpreted and 9p21 and centromere 9 copy numbers were valued for each tissue spot. The presence of approximately the same copy numbers of 9p21 and centromere 9 in tumor cell nuclei was assumed as normal. Heterozygous 9p21 deletion was assumed if fewer 9p21 than centromere 9 signals in ≥60% of all tumor cell nuclei or only one 9p21 and one centromere 9 signal (monosomy of chromosome 9) in nearly all tumor cell nuclei were counted. The presence of centromere 9 signals in the case of a complete lack of 9p21 signals in the tumor cell nuclei was considered a homozygous 9p21 deletion if unequivocal 9p21 and centromere 9 signals in tumor-adjacent normal cell nuclei were present. Tissue spots lacking 9p21 signals in all (tumor and normal cell) nuclei were considered as not evaluable and were excluded from the analysis.

### 2.3. Immunohistochemistry (IHC)

IHC was performed as previously described [21]. Freshly prepared TMA sections (2.5 µm cut thickness) were immunostained in one experiment on one day in a Dako Omnis automated stainer (Agilent Technologies, Santa Clara, CA, USA) using the EnVision FLEX, High-pH Kit (Agilent Technologies, Santa Clara, CA, USA, #GV800). Tissues were deparaffinized with Clearify^TM^ agent (Agilent Technologies, Santa Clara, CA, USA, #GC810) and exposed to heat-induced antigen retrieval at 97 °C for 30 min in Target Retrieval Solution, High-pH reagent (part of the Agilent kit #GV800). The primary antibody specific for MTAP (recombinant rabbit monoclonal, MSVA-741R, MS Validated Antibodies GmbH, Hamburg, Germany, #5293-741R) was applied at a dilution of 1:50 for 30 min at ambient temperature. Blocking of endogenous peroxidase activity was performed with Peroxidase Blocking Reagent (part of the Agilent kit #GV800) for 3 min. The bound antibody was visualized with the EnVision FLEX, High-pH Kit reagents DAB+ Chromogen and Substrate Buffer (parts of the Agilent kit #GV800) and EnVision FLEX + Rabbit LINKER (Agilent Technologies, Santa Clara, CA, USA; #GV809) according to the manufacturer’s instructions. The tissue spots were counterstained with hemalaun. For cancer tissues, the average staining intensity of unequivocally neoplastic cells was estimated as 0, 1+, 2+, and 3+. For classification of a cancer as completely negative (0), the presence of unequivocal MTAP staining in cancer-adjacent normal tissue cells was required. Tissues with a complete absence of MTAP staining in cancerous cells and a lack of stromal cells with unequivocal MTAP staining were considered “non-informative” and excluded from the analysis.

### 2.4. Whole-Section Validation and Heterogeneity Analysis

All tumor-containing blocks from 7 cancers analyzed on the heterogeneity TMA with either heterogeneous or questionable MTAP staining were subjected to whole-section MTAP IHC. To determine whether small areas with discrepant/heterogenous MTAP status had remained unidentified in our TMA analysis, all available tissue blocks of 19 consecutive additional pancreatic adenocarcinomas were subjected to IHC analysis using protocols identical to those for the TMAs. This analysis involved a total of 135 whole sections from 19 cancers (average: 7 slides per patient; minimum: 2, maximum: 14).

### 2.5. Statistics

Statistical calculations were performed with JMP17^®^ software (SAS^®^, Cary, NC, USA). Contingency tables and the chi² test were performed to search for associations between MTAP immunostaining and tumor phenotype as well as 9p21 copy number status. ANOVA analysis was used to search for associations between MTAP immunostaining and tumor size.

## 3. Results

### 3.1. Technical Results

Stainings were informative for IHC in 478 (62.2%) and for FISH in 423 (55.0%) of the 769 arrayed primary pancreatic ductal adenocarcinoma samples, and in 901 (57.1%; IHC) or 698 (44.2%; FISH) of the 1578 arrayed samples of the heterogeneity TMA. The remaining 968 (IHC)/1226 (FISH) tissue samples were not interpretable because of a lack of unequivocal tumor cells, absence of MTAP staining in both cancerous and normal tissues, insufficient hybridization (FISH), or a lack of the entire tissue spot in the TMA section.

### 3.2. MTAP Deficiency in Primary Tumors

**Figure 1:**
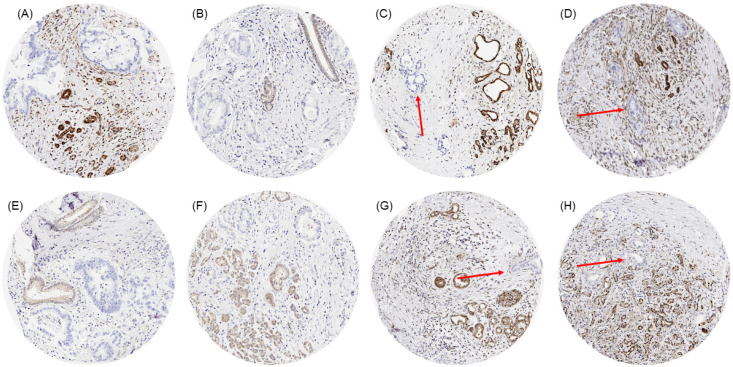
MTAP immunostaining in primary pancreatic ductal adenocarcinoma with small (**A**,**B**,**E**,**F**) and very small areas (**C**,**D**,**G**,**H**; red arrows) of neoplastic glands without any MTAP immunostaining next to non-neoplastic glands and stroma cells with distinct MTAP immunostaining.

A complete absence of MTAP staining was seen in 181 (37.9%) of 478 IHC-interpretable TMA samples, while 143 (29.9%) showed 1+, 97 (20.3%) showed 2+, and 57 (11.9%) showed 3+ MTAP staining. According to the stroma-rich nature of many pancreatic adenocarcinomas and the frequently large distance between individual tumor glands, many samples contained only a few small tumor glands. Especially in low-grade tumors, MTAP deficiency often facilitated the distinction of neoplastic from non-neoplastic glands. Representative images of MTAP immunostaining are shown in Figure 1. MTAP deficiency (Table 1) and the level of MTAP expression (Supplementary Table S2) were both unrelated to pT, pN, grade, tumor size, and resection margin status in our 478 interpretable primary pancreatic adenocarcinomas and unrelated to overall survival in a subset of 64 primary pancreatic adenocarcinomas (Supplementary Figure S1).

### 3.3. MTAP Heterogeneity

**Figure 2:**
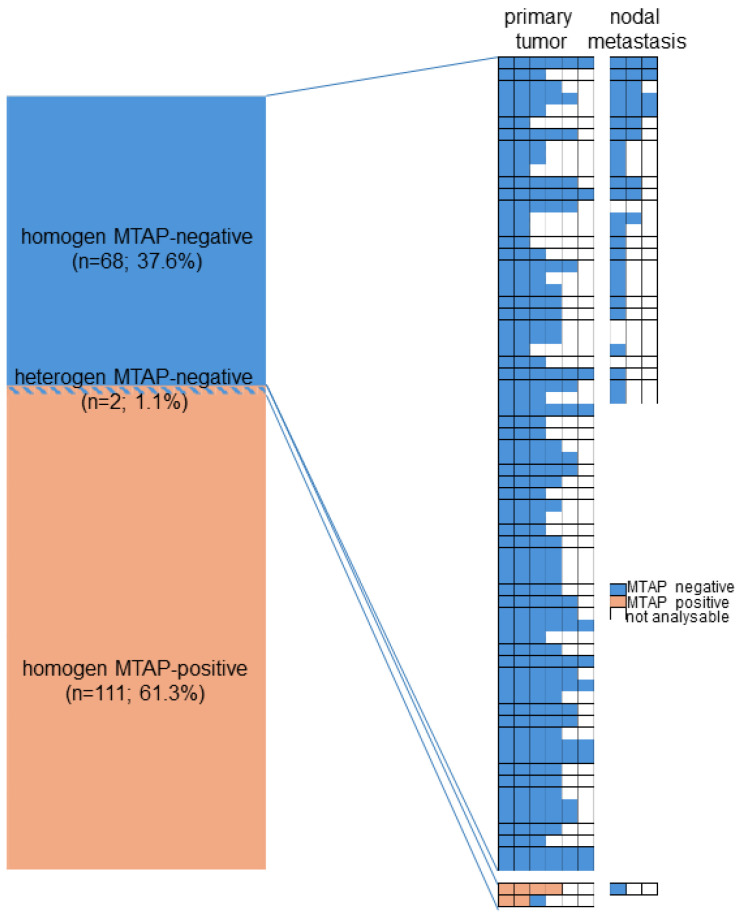
Heterogeneity of MTAP immunostaining.

**Figure 3:**
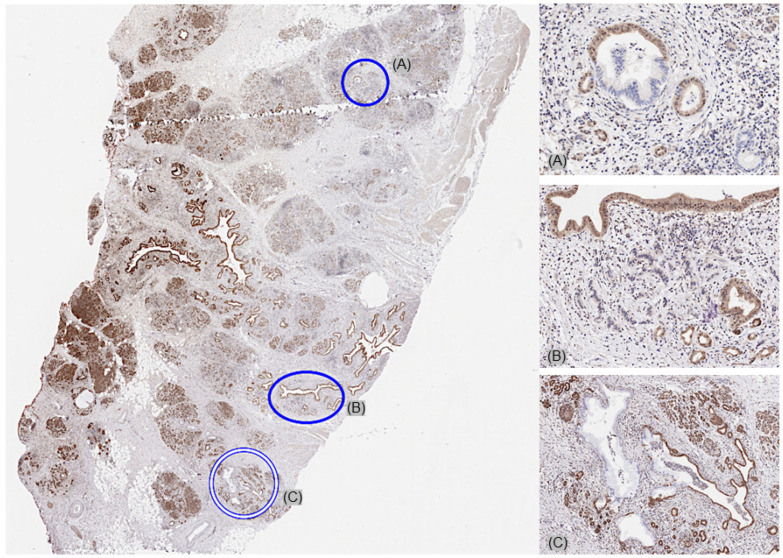
Large-section validation revealed a heterogeneity of MTAP staining in a pancreatic intraepithelial neoplasia (PanIN) (**A**,**C**), while the adjacent invasive cancer was homogeneously MTAP-deficient (**B**).

**Figure 4:**
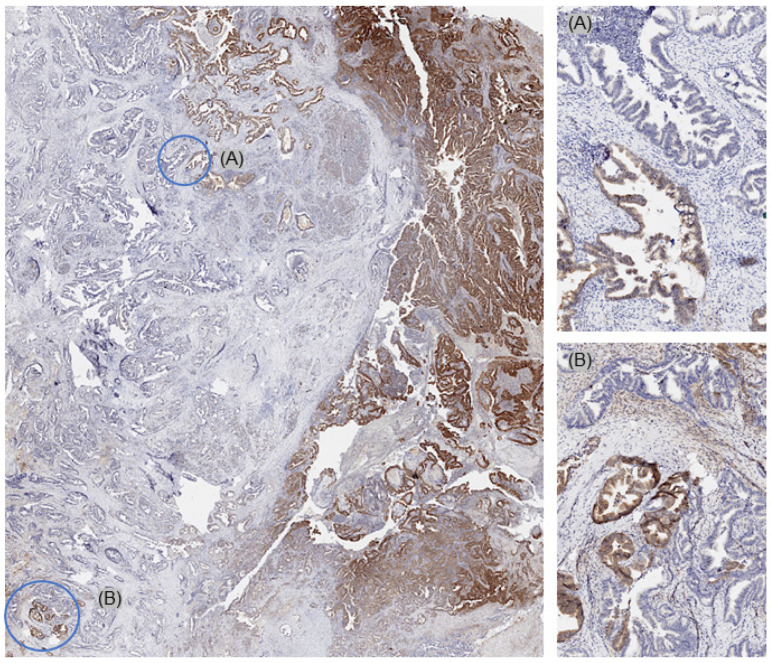
Whole-section validation led to the identification of one pancreatic adenocarcinoma with heterogeneous MTAP deficiency. This cancer showed large areas with both MTAP-deficient (**A**) and MTAP-proficient (**B**) cancer areas.

On a patient level, MTAP deficiency was found in 70 (38.7%) of 181 tumors, for which at least three different samples were interpretable on our set of heterogeneity TMAs (Figure 2). Of these patients, 68 (97.1%) showed an MTAP loss in all interpretable samples (homogeneous MTAP deficiency) and one showed a heterogeneous MTAP deficiency. In one additional case, the large-section validation revealed a heterogeneity of MTAP staining in a pancreatic intraepithelial neoplasia (PanIN) of the pancreas, while the adjacent invasive cancer was homogeneously MTAP-deficient (Figure 3). The additional whole-section evaluation of 19 consecutive cases led to the identification of one pancreatic adenocarcinoma with heterogeneous MTAP deficiency, while 4 cases showed a homogeneous MTAP deficiency, and 14 cases homogeneously retained MTAP expression. The cancer with heterogeneous MTAP status contained large areas of both MTAP-deficient and MTAP-proficient adenocarcinoma (Figure 4).

### 3.4. MTAP FISH vs. IHC Results

**Figure 5:**
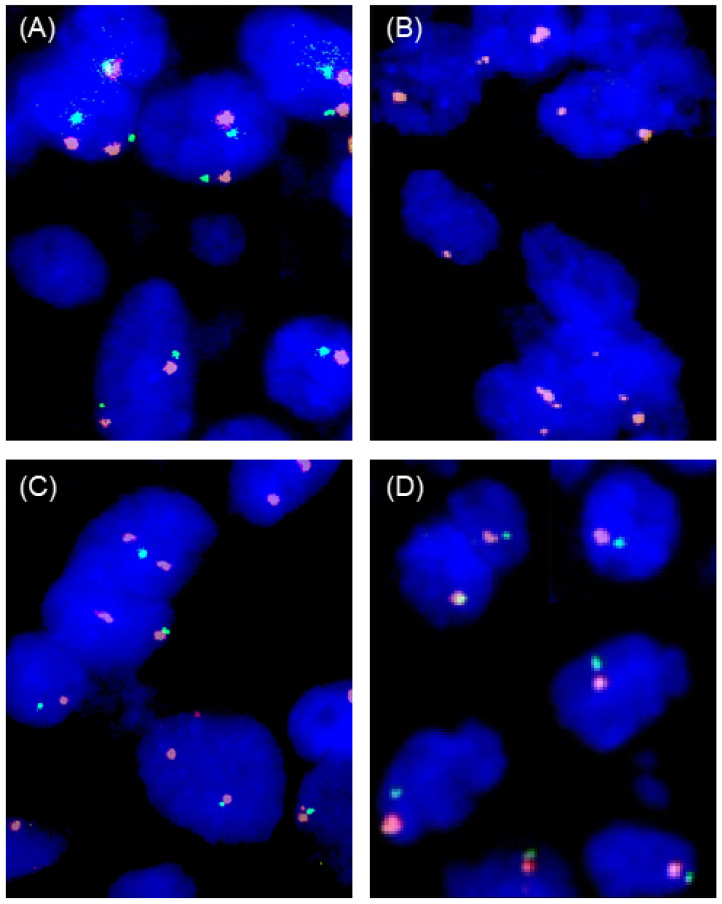
Examples of FISH findings. (**A**) Normal 9p21 copy number status with two green 9p21 signals and two orange centromere 9 signals, (**B**) homozygous 9p21 deletion without any green 9p21 signals in the tumor nuclei but presence of orange centromere 9 signals, (**C**) heterozygous 9p21 deletion with fewer green 9p21 signals than orange centromere 9 signals, and (**D**) chromosome 9 monosomy with one green 9p21 and one orange centromere 9 signal.

**Figure 6:**
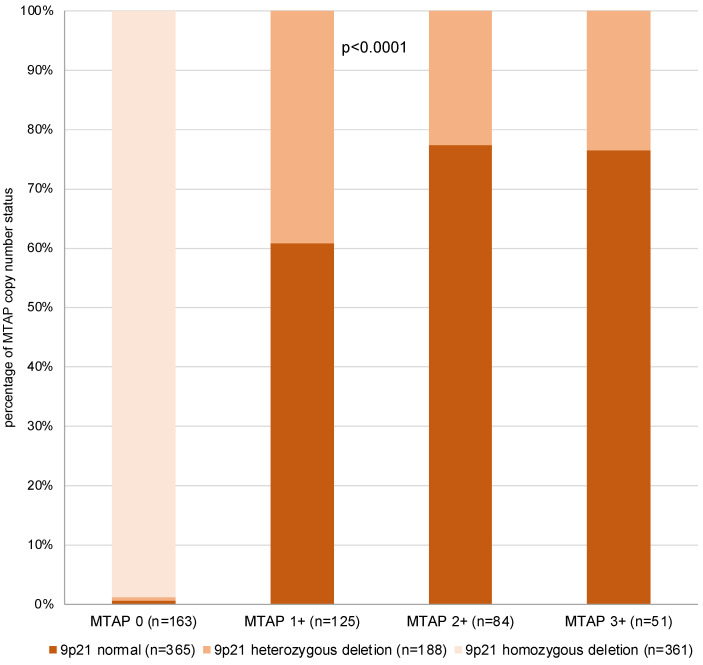
MTAP immunostaining vs. 9p21 copy number status.

Among 423 interpretable primary tumors, a homozygous 9p21 deletion was seen in 161 (38.1%) and a heterozygous deletion in 81 (19.1%) of cases. Examples of FISH findings are shown in Figure 5. There was a near-perfect correlation between IHC and FISH data (*p* < 0.0001, Figure 6). Almost all of the 163 samples (n = 161; 98.8%) with a complete MTAP expression loss and available FISH data had a homozygous 9p21 deletion while there were no cases with homozygous deletions within the 260 samples with retained MTAP expression (99.5% concordance). Heterozygous deletions were seen in 49 (39.2%) of the 125 samples with 1+ staining but in only 19 (22.6%) of the 84 samples with 2+ staining and 12 (23.5%) of the 51 samples with 3+ positivity staining by IHC (*p* < 0.0001).

## 4. Discussion

These data reveal a high prevalence (>35%) of MTAP deficiency in ductal adenocarcinoma of the pancreas and show that MTAP deficiency is mostly homogeneous across the entire cancer. Our findings also suggest a considerable diagnostic utility of MTAP IHC for the detection of pancreatic adenocarcinoma, especially in the case of low-grade cancer and in small biopsies.

The results of this study identify 37.9% of our primary ductal adenocarcinomas of the pancreas as MTAP-deficient. This number is somewhat higher than the 29.7% in our previous study analyzing a subset of 531 of our 769 primary ductal adenocarcinomas in a comparative evaluation of 149 different tumor entities for MTAP deficiency [18]. This difference in the rate of MTAP deficiency is probably due to a more rigorous exclusion of samples lacking unequivocal cancer tissue in this study. Other studies had reported MTAP deficiency by IHC in 27–30% [22,23,24] and deep deletions by next-generation sequencing (NGS) in 2–21% of ductal adenocarcinomas of the pancreas [25,26]. The somewhat lower rates of MTAP deficiency in NGS studies may be due to the notoriously low tumor cell density in pancreatic adenocarcinomas, which are characterized by a dense desmoplastic stroma and large distances between invasively growing tumor cell glands. As individual tumor glands may appear deceptively benign, the recognition of pancreatic adenocarcinoma is often challenging on small biopsies from low-grade tumors. That a complete MTAP expression loss can greatly facilitate the affirmative diagnosis of an intraductal or invasive pancreatic neoplasm is also supported by data from Yu et al. [22]. These authors identified a complete MTAP expression loss in 4 of 21 cases classified as suspicious and in 4 of 22 cases classified as atypical in a cohort of 136 EUS-FNA cell blocks or core biopsies targeting solid pancreatic masses and reported that subsequent surgery confirmed MTAP-deficient invasive cancer in all 8 cases that were morphologically equivocal and MTAP-deficient [22].

The successful comparative analysis of 423 primary pancreatic adenocarcinoma samples by FISH and IHC on consecutive sections demonstrated a high precision of MTAP IHC for the detection of cases with a homozygous *MTAP* deletion. This is in line with previous studies founding sensitivities between 59% and 100% and specificities between 96% and 100% for detection of homozygous deletion by MTAP IHC in urothelial bladder carcinomas, mesothelioma, astrocytoma, and meningioma (summarized in [18]). In addition, our assay had earlier been validated according to the recommendations of the International Working Group for Antibody Validation (IWGAV) by comparison with an independent second antibody in 76 different normal tissues [18,27]. The staining conditions had been titrated by using a TMA composed of 20 wild-type cells and 20 heterozygously and 20 homozygously *MTAP*-deleted bladder cancers to obtain maximal staining intensity in wild-type cells while retaining a complete absence of background staining in MTAP-deficient cancers [21]. The near-perfect correlation between MTAP deficiency by IHC and homozygous deletion by FISH confirms the assay quality and characterizes ductal adenocarcinomas of the pancreas as a tumor entity where MTAP deficiency is always caused by deletion. We have previously shown that such a near-complete concordance between MTAP IHC and FISH analysis does not apply to all tumor entities. In contrast to pancreatic adenocarcinomas, MTAP deficiency usually occurs in the absence of 9p21 deletions in malignant lymphomas and neuroendocrine neoplasms [18]. The significant association between low MTAP expression and heterozygous *MTAP* deletions indicates that MTAP expression is gene-dosage-dependent in pancreatic cancer. This is in line with the finding that—at the genetic level—large 9p21 deletions are the main mechanism for MTAP inactivation, whereas MTAP gene mutations are rather rare [28]. However, slight variations in the results may be also explained by epigenetic factors, such as DNA methylation and histone deacetylation [29]. However, a more quantitative measurement of MTAP immunostaining and the use of non-neoplastic tissue as a reference value to compensate for the impact of preanalytical variabilities could potentially enable the detection of heterozygous *MTAP* deletions by IHC.

The high rate of homogeneous MTAP deficiency in ductal adenocarcinomas of the pancreas is of clinical importance. Our finding suggests that (a) the detection of MTAP deficiency in a small pancreatic biopsy will be representative of the entire tumor in the vast majority of cases, and (b) if drugs targeting MTAP-deficient cancers should be introduced into clinical practice the entire tumor mass should be amenable to these drugs in most patients. MTAP deficiency in pancreatic adenocarcinoma appears to be a suitable target of new therapies and trial approaches under development [30,31,32]. The fact that only 1 of 19 (5.3%) consecutive cancers analyzed for MTAP expression on all tumor-containing whole sections and 1.1% of 181 cases analyzed on our heterogeneity TMA showed heterogeneous MTAP deficiency suggests that homozygous 9p21 deletion either occurs early in the development of affected pancreatic cancers or provides a growth advantage to affected cells that enables a rapid overgrowth of clones lacking this deletion. The complete absence of associations between MTAP deficiency and parameters of aggressive disease in our 478 primary ductal pancreatic adenocarcinomas argues against the increased aggressiveness of MTAP-deficient cancer clones, although Jiang et al. [26] recently found a link between 9p21 deletion and poor prognosis in a cohort of 48 pancreatic adenosquamous cancers.

The high rate of homogeneous MTAP deficiency in ductal adenocarcinomas of the pancreas is of potential clinical significance. Earlier data from us and others had demonstrated that the rate of heterogeneous cases varies considerably between both the type of alteration and tumor entities. For example, high homogeneity rates had been found for expression loss of mismatch repair genes or microsatellite instability across several tumor entities including colorectal [33], ovarian [34], and pancreatic [35] carcinomas. Heterogeneity of *HER2* amplification depends on the cancer type, however. While heterogeneity of high-level *HER2* amplification and overexpression is very low (up to 11%) in breast cancer [36], it is moderate (approximately 50%) in gastric adenocarcinoma [37] and (approximately 50%) in urothelial carcinoma [38]. A high rate of intratumoral heterogeneity is also common for *ALK* rearrangements in lung cancer [39], *PTEN* deletion in prostate cancer [40], as well as *BRAF* mutation in pulmonary adenocarcinoma [41].

## 5. Conclusions

Loss of MTAP expression is frequent, mostly homogenous, and always caused by homozygous 9p21 deletion in pancreatic ductal adenocarcinomas. Due to the aggressive clinical behavior of pancreatic adenocarcinomas, this tumor type may represent an ideal cancer type for clinical trials studying new drugs targeting MTAP-deficient cancer cells.

## Figures and Tables

**Table 1:** MTAP immunostaining and tumor phenotype.

Pathological Parameters			MTAP Status (%)		
		n	Loss	Retained	*p*-Value
Analyzable tumors		478	37.9	62.1	
Tumor stage	pT1	11	54.6	45.5	0.6462
	pT2	72	40.3	59.7	
	pT3	354	37.0	63.0	
	pT4	37	35.1	64.9	
Nodal stage	pN0	111	39.6	60.4	0.5302
	pN+	336	36.3	63.7	
Grade	G1	21	33.3	66.7	0.7951
	G2	303	38.3	61.7	
	G3	127	35.4	64.6	
Tumor size (mm)	mean ± SD	347	38.4 ± 1.8	39.1 ± 1.4	0.7575

## Data Availability

All data generated or analyzed during this study are included in this published article.

## Supplementary Materials

The following supporting information can be downloaded at https://www.mdpi.com/article/10.3390/cancers17071205/s1, Table S1: Patient cohort; Table S2: MTAP immunostaining intensity and tumor phenotype; Figure S1: MTAP immunostaining and overall survival
