# Supplementary material for: Deficiency of MTAP Is Frequent and Mostly Homogeneous in Pancreatic Ductal Adenocarcinomas
Source: Cancers (Basel). 2025 Apr 1;17(7):1205. doi: 10.3390/cancers17071205 (PMC11987894; doi:10.3390/cancers17071205)
Supplement: Supplementary file 1 [file cancers-17-01205-s001.zip › cancers-3485112-supplementary/Supplementary Table S1.pdf]

Pathological parameters		patients on the TMA (n=769)	
		n	%
Tumor stage	pT1	22	2.9
	pT2	110	14.4
	pT3	574	75.2
	pT4	57	74.7
Nodal stage	pN0	184	25.2
	pN+	545	74.8
Grade	G1	29	4.0
	G2	511	69.8
	G3	191	26.1

Percent in the column "patients on the TMA" refers to the fraction of samples across each category. Numbers do not always add up to 769 in the different categories because of cases with missing data.
