# Supplementary material for: Deficiency of MTAP Is Frequent and Mostly Homogeneous in Pancreatic Ductal Adenocarcinomas
Source: Cancers (Basel). 2025 Apr 1;17(7):1205. doi: 10.3390/cancers17071205 (PMC11987894; doi:10.3390/cancers17071205)
Supplement: Supplementary file 1 [file cancers-17-01205-s001.zip › cancers-3485112-supplementary/Supplementary Table S2.pdf]

Pathological parameters		MTAP status (%)					
		n	negative	1+	2+	3+	p value
analyzable Tumors		478	37.9	29.9	20.3	11.9	
Tumor stage	pT1	11	54.6	9.1	18.2	18.2	0.5498
	pT2	72	40.3	27.8	23.6	8.3	
	pT3	354	37.0	32.2	19.2	11.6	
	pT4	37	35.1	21.6	27.0	16.2	
Nodal stage	pN0	111	39.6	24.3	24.3	11.7	0.3376
	pN+	336	36.3	32.7	19.1	11.9	
Grade	G1	21	33.3	14.3	47.6	4.8	0.1219
	G2	303	38.3	30.0	20.1	11.6	
	G3	127	35.4	33.1	18.1	13.4	
Tumor size (mm)	mean ± SD	347	38.4 ±1.8	39.0 ±1.9	39.1 ±2.5	39.2 ±3.7	0.9922
