# Supplementary figures and images for: Deficiency of MTAP Is Frequent and Mostly Homogeneous in Pancreatic Ductal Adenocarcinomas
Source: Cancers (Basel). 2025 Apr 1;17(7):1205. doi: 10.3390/cancers17071205 (PMC11987894; doi:10.3390/cancers17071205)

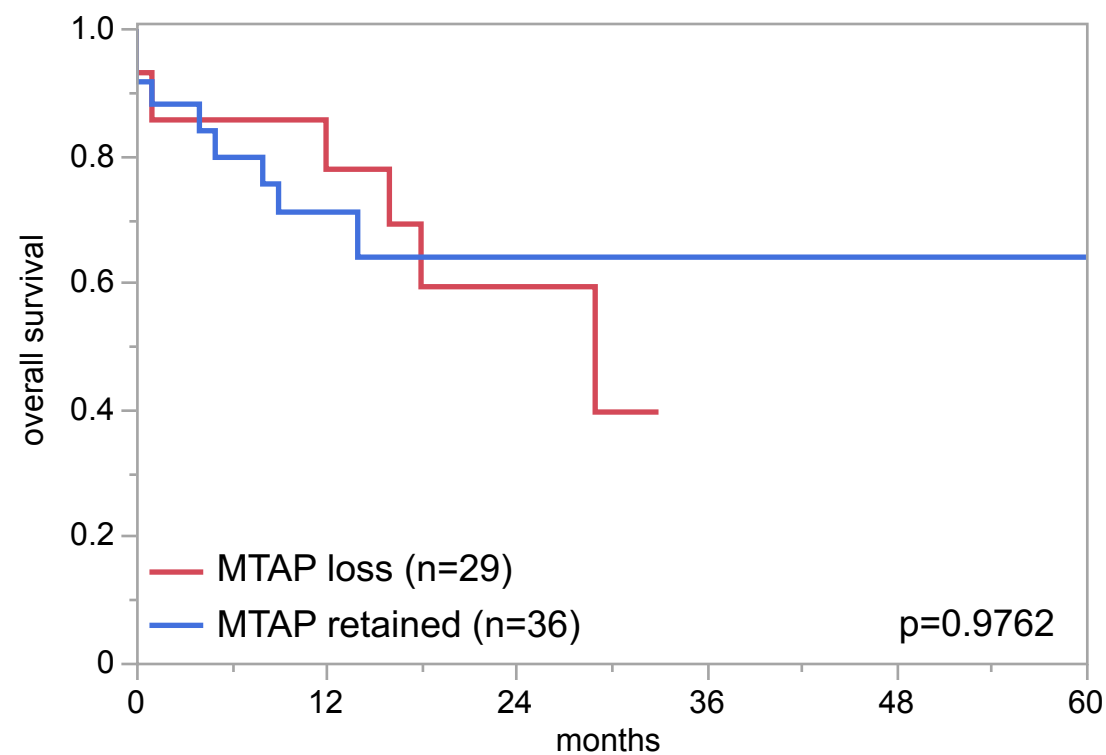

Supplement: Supplementary file 1 [file cancers-17-01205-s001.zip › cancers-3485112-supplementary/Supplement Figure S1.pdf]
